# False Mating of Blackbirds (*Turdus merula*) and Fieldfares (*Turdus pilaris*)

**DOI:** 10.1002/ece3.70663

**Published:** 2025-01-18

**Authors:** Dariusz Wysocki, Joanna Dudzińska‐Nowak

**Affiliations:** ^1^ Institute of Marine and Environmental Science, University of Szczecin Szczecin Poland

**Keywords:** breeding behavior, Turdidae, *Turdus merula*, *Turdus pilaris*

## Abstract

During an intensive study of the urban population of the blackbird *Turdus merula* in Szczecin (1997–2023), four cases of surrogate copulation were observed in the blackbird and three in the fieldfare *Turdus pilaris* . In three cases, birds (blackbirds twice and fieldfare once) try to copulate with fledglings of their own species, and in all other cases, the birds try to copulate with moss and sticks. In three cases copulation, attempts were done by adult birds (two blackbirds and one fieldfare), and in four cases, it was a fledgling.

## Graphical abstract

The article describes seven surrogate copulations observed in blackbirds and fieldfares in light of hypotheses explaining this phenomenon.

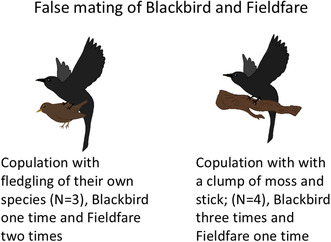

## 1 Introduction

Copulatory behavior in birds, its causes and consequences, is relatively well known (see Birkhead and Møller 1992 for review and Wysocki and Halupka 2004 for blackbirds), but the reason for copulation with surrogate objects is still unknown, despite being observed in many bird species (Simon 1940; Young 1949; Ficken and Dilger 1960; Sordahl 2001). According to Hamilton (1975), such copulations should be expected to be more frequent at the beginning of the breeding season, the objects of sexual interest should be similar in size to the female and are a substitute for females in the case of an excess of males in relation to sexually receptive females.

Here we present the description of four cases of surrogate copulation in the blackbird *Turdus merula* and three in the fieldfare *Turdus pilaris* observed during an intensive study of the urban population of the blackbird *Turdus merula* in Szczecin (NW Poland).

## 2 Study Area and Data Collection

Data were collected during a long‐term study (1997–2024) of a blackbird population breeding in an urban park in Szczecin (NW Poland, 53°26′ N, 14°34′ E). Every year since 2000, over 90% of this population has been color‐ringed during the breeding season (March–August) so we know the exact age of most blackbirds. In the case of fieldfare, the exact age was unknown. For detailed method and study area description, see Jankowiak, Wysocki, and Greño (2016).

## 3 Results and Discussion

We observed seven cases of false copulation cases in total in the past 17 years of field observation (Table 1 summarizes all observations of the surrogate copulation). In three cases, birds (twice blackbird and once a fieldfare) try to copulate with a fledgling of their own species, and in all other cases, the birds try to copulate with moss and sticks. In three cases, copulation attempts were done by adult birds (two blackbirds and one fieldfare). In four cases, the copulating birds were fledglings or juveniles. The first three copulations with surrogate objects (one from 2000 and two from 2001) have already been described (Wysocki 2004), but due to the extremely rare observation of such behavior, and that publication being in Polish, we decided to include those observations in this article.

**TABLE 1:** Surrogate copulations in blackbirds and fieldfare.

Species	Age	Data	Object of surrogate copulation
Blackbird	1	29 July, 2000	One copulation with a stick
Blackbird	2	31 May, 2001	Six copulations with 16 days old blackbird fledgling
Fieldfare	1	10 June, 2001	One copulation with fieldfare fedgling
Blackbird	2	10 June, 2004	Three copulations with a clump of moss
Fieldfare	Adult > 1 year	15 June, 2006	One copulation with a fieldfare fledgling
Fieldfare	1	05 June, 2018	One copulation with a stick
Blackbird	1	19 June, 2018	Three copulations with a stick

On July 29, 2000, a 3‐month‐old blackbird tried to copulate with a branch lying on the ground—it jumped on the stick characteristically flapping its wings and lowering its rump in exactly the same way as during typical copulation. The second observed case occurred on May 31, 2001 when a male in its second calendar year of life tried to copulate with a 16‐day‐old blackbird fledgling. He jumped on the chick's back six times, performing movements characteristic of copulation. A similar situation occurred on June 10, 2001 when a fieldfare tried to mate with a conspecific juvenile, behaving in exactly the same way as the previously described blackbird. On June 10, 2004, an adult blackbird (in his second calendar year of life) tried to copulate with a clump of moss three times. He behaved in a similar way to the previously described case of false copulation with a stick. On June 15, 2006, an adult fieldfare tried to copulate with a fledgling, and on June 5, 2018, just after a fight with a blackbird juvenile, a fieldfare juvenile tried to copulate with a stick. The last observation took place on June 19, 2018 when a blackbird juvenile tried to copulate with a stick three times.

The sexual behavior of blackbirds is relatively easy to observe. We estimated the number of copulations per pair in blackbirds at 24.2 in a single reproductive cycle (Wysocki and Halupka 2004), but the average number of observed copulations in the case of blackbirds is approximately 10 per year. Forced extra pair copulation (FEPC) in the studied population (Wysocki et al. in preparation) is easier to observe—we observe about 20 FEPC cases per year. In fieldfares, copulation and FEPC are relatively common but because we focus on the population of color‐ringed blackbirds, our knowledge is less detailed. It can be assumed that the number of copulations and FEPC in fieldfares are similar to those observed in blackbirds, so the observed number of false copulation indicates that it is an extremely rare phenomenon in these species.

As the blackbird breeding season length depends on spring temperature and summer precipitation (Jankowiak, Pietruszewska, and Wysocki 2014), the studied population usually starts to breed in March (the first brood was observed on 25 February, 2020) and ends in July (the last brood was found on 25 July, 2016). In the case of fieldfare, the first broods appear in April and the last ones in May or at the beginning of June (Wysocki personal observation). So, all observed surrogate copulations took place in the second part of the breeding season, which is opposite to Hamilton's (1975) prediction. In three cases, the object (fledgling) was similar to a female, but in four cases (stick and moss), it was not. Also, the suggestion that surrogate copulation takes place in the situation of an excess of males in relation to sexually receptive females is not in line with most of our observations. In four out of seven observations, a bird trying to copulate with surrogate objects involved individuals that were immature or not yet reproductively viable (fledgling). On the other hand, three surrogate copulations were observed in adult birds at the end of the breeding season, when most blackbird and fieldfare females are not sexually receptive, as Hamilton predicted.

It seems that the best explanation for the causes of observed false copulation is the presence of a “mating filter,” which describes the breadth of stimuli that will elicit sexual behaviors (Richardson and Zuk 2023). It is not known what factors influence the breadth of the mating filter. On the basis of our observations, we can speculate that the maturity of the reproductive system and stress (caused both by interactions with other individuals and by the lack of a sexual partner) can affect it. In most cases, juveniles were involved in surrogate copulation, therefore, we cannot exclude that the observed behavior is connected with maturation of the juvenile (with a broad mating filter) reproductive system. In this case, maturation will be connected with a narrowing of the mating filter. Similarly, the fieldfare juvenile copulation with a twig just after the end of a fight with a blackbird juvenile may suggest that false copulation could be a result of elevated stress (Weaver and Williams 2016) and relieving tension resulting from this fight. The lack of a proper sexual partner could make the mating filter broader (both male adult blackbirds involved in false copulation probably did not have a partner during the breeding season, in the case of fieldfares we have no such information). It cannot be ruled out that the observed juveniles' behavior was adaptive due to the benefits of training. Copulation in birds demands some skill, for example, in the Kittiwake *Rissa tridactyla* , a higher proportion of copulation without cloacal contact was found in birds breeding together for the first time (Chardine 1987). We also cannot exclude that in the case of juvenile birds, the observed behavior is a result of social learning (see Zenatal 2004 for review). In the case of adult blackbirds and fieldfares, we can only speculate. Two of the hypotheses explaining the occurrence of masturbation in animals (Brindle et al. 2023) may also explain the occurrence of false mating in adult blackbirds and fieldfares. False mating could be a byproduct of high underlying sexual arousal, or according to the Sperm Quality Hypothesis (Baker and Bellis 1993), false mating may expel inferior sperm, improving subsequent ejaculate quality. Because all three false copulations were observed at the end of the breeding season, the first explanation seems to us more probable.

## Author Contributions

**Dariusz Wysocki:** conceptualization (lead), data curation (lead), formal analysis (lead), funding acquisition (equal), investigation (lead), methodology (lead), project administration (lead), resources (equal), writing – original draft (equal). **Joanna Dudzińska‐Nowak:** funding acquisition (equal), writing – original draft (equal).

## Conflicts of Interest

The authors declare no conflicts of interest.

## Data Availability

All data are included in Table 1.

## References

[ece370663-bib-0001] Baker, R. R. , and M. A. Bellis . 1993. “Human Sperm Competition: Ejaculate Adjustment by Males and the Function of Masturbation.” Animal Behaviour 46: 861–885. 10.1006/anbe.1993.1271.

[ece370663-bib-0002] Birkhead, T. R. , and A. P. Møller . 1992. Sperm Competition in Birds. Evolutionary Causes and Consequences. London, UK: Academic Press.

[ece370663-bib-0003] Brindle, M. , H. Ferguson‐Gow , J. Williamson , R. Thomsen , and V. Sommer . 2023. “The Evolution of Masturbation Is Associated With Postcopulatory Selection and Pathogen Avoidance in Primates.” Proceedings of the Royal Society B 290: 20230061. 10.1098/rspb.2023.0061. 37282530 PMC10244968

[ece370663-bib-0004] Chardine, J. W. 1987. “The Influence of Pair‐Status on the Breeding Behaviour of the Kittiwake *Rissa tridactyla* Before Egg‐Laying.” Ibis 129: 515–526.

[ece370663-bib-0005] Ficken, M. S. , and W. C. Dilger . 1960. “Comments on Redirection With Examples of Avian Copulations With Substitute Objects.” Animal Behaviour 8: 219–222.

[ece370663-bib-0006] Hamilton, R. B. 1975. “Comparative Behavior of the American Avocet and the Black‐Necked Stilt (Recurvirostridae).” Ornithological Monographs 17: iii‐98.

[ece370663-bib-0007] Jankowiak, Ł. , H. Pietruszewska , and D. Wysocki . 2014. “Weather Conditions and Breeding Period Duration of Blackbird ( *Turdus merula* ).” Folia Zoologica 63, no. 4: 245–250.

[ece370663-bib-0008] Jankowiak, Ł. , D. Wysocki , and J. Greño . 2016. “Survival and Site Fidelity of Urban Blackbirds *Turdus merula* —Comparison of Cormack‐Jolly‐Seber and Barker Models.” Acta Ornithologica 51: 189–197. 10.3161/00016454AO2016.51.2.005.

[ece370663-bib-0009] Richardson, J. , and M. Zuk . 2023. “Rethinking Same‐Sex Sexual Behaviour: Male Field Crickets Have Broad Mating Filters.” Proceedings of the Royal Society B 290: 20230002. 10.1098/rspb.2023.0002. 37122255 PMC10130708

[ece370663-bib-0010] Simon, J. R. 1940. “Mating Performance of the Sage Grouse.” Auk 57: 467–471.

[ece370663-bib-0011] Sordahl, A. 2001. “Copulatory Behavior of American Avocets and Black‐Necked Stilts.” Auk 118: 1072–1076. 10.2307/4089862.

[ece370663-bib-0012] Weaver, P. G. , and B. W. Williams . 2016. “Observations of False Mating Behavior in Entocytherid Ostracods From the Northwestern United States.” Invertebrate Biology 135, no. 3: 252–258. 10.1111/ivb.12141.

[ece370663-bib-0013] Wysocki, D. 2004. “Observations of Surrogate Copulations of a Blackbird ( *Turdus merula* ) and Fieldfare ( *Turdus pilaris* ).” Notatki Ornitologiczne 45: 124–125.

[ece370663-bib-0014] Wysocki, D. , and K. Halupka . 2004. “The Frequency and Timing of Courtship and Copulation in Blackbirds, *Turdus Merula* , Reflect Sperm Competition and Sexual Conflict.” Behaviour 141, no. 4: 501–512. 10.1163/156853904323066766.

[ece370663-bib-0015] Young, H. 1949. “Atypical Copulatory Behaviour of a Robin.” Auk 66: 94.

[ece370663-bib-0016] Zenatal, T. R. 2004. “Action Imitation in Birds.” Learning & Behavior 32, no. 1: 15–23. 15161137 10.3758/bf03196003

